# Step‐by‐Step Technique for Platelet‐Rich Plasma Gel Preparation and Use in Knee Osteoarthritis

**DOI:** 10.1002/atn2.70133

**Published:** 2026-05-24

**Authors:** Diego Ariel de Lima, Renata Clazzer, Carlos Eduardo da Silveira Franciozi, Ávila Kelly de Medeiros Nicolau, Isadora Pereira Bandeira, Lana Lacerda de Lima, Maria Luzete Costa Cavalcante

**Affiliations:** ^1^ UFERSA, Universidade Federal Rural do Semi‐Árido Mossoró Brasil; ^2^ UNIFESP ‐ Universidade Federal de São Paulo São Paulo Brazil; ^3^ UFC, Departamento de Cirurgia da Universidade Federal do Ceará Fortaleza Brazil

## Abstract

Knee osteoarthritis (OA) remains a leading cause of pain and disability worldwide, and current injectable options such as corticosteroids and hyaluronic acid often provide limited or short‐term benefits. Orthobiologic therapies, particularly platelet‐rich plasma (PRP), have gained attention for their regenerative and anti‐inflammatory potential. PRP plasma gel is a autologous formulation obtained through controlled thermal denaturation of plasma proteins, producing an albumin‐based semisolid matrix subsequently mixed with fresh PRP. This process results in a homogeneous gel capable of sustained release of bioactive growth factors, potentially prolonging intra‐articular residence time and clinical effect compared with conventional PRP. We describe a step‐by‐step technique for preparing PRP plasma gel using standard laboratory equipment, without the need for commercial kits. The method is reproducible, low cost, and suitable for outpatient settings, including resource‐limited environments. Clinical application is shown in knee osteoarthritis, where the formulation can be injected intra‐articularly as an accessible and versatile orthobiologic option. The technique offers technical and economic advantages, with the potential to broaden the use of orthobiologic therapies in daily practice.

## Graphical abstract

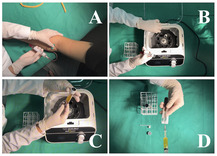

**VIDEO 1:** Step‐by‐step technique for platelet‐rich plasma (PRP) gel preparation and use in knee osteoarthritis. The video shows (1) peripheral blood collection and centrifugation, (2) formation of the albumin gel by controlled heating, (3) mixing with fresh PRP using a three‐way connector to obtain a homogeneous semisolid formulation, and (4) intra‐articular injection of the final PRP plasma gel into the knee joint. Video content can be viewed at https://doi.org/10.1002/atn2.70133.

Knee osteoarthritis (OA) is a highly prevalent degenerative joint disease and one of the leading causes of pain, disability, and reduced quality of life worldwide. Conservative treatment options, including weight reduction, physical therapy, nonsteroidal anti‐inflammatory drugs, and intra‐articular injections of corticosteroids or hyaluronic acid, often provide only limited or temporary relief. Consequently, there is growing interest in orthobiologic therapies that may promote tissue regeneration and modulate inflammation more effectively.^1^, ^2^, ^3^

Platelet‐rich plasma (PRP) has been widely investigated as a minimally invasive and autologous option for the management of knee OA. However, conventional liquid PRP formulations are rapidly resorbed from the joint space, limiting their therapeutic duration. To overcome this limitation, PRP‐derived formulations have been developed to enhance intra‐articular persistence and optimize the sustained release of growth factors.^4^, ^5^, ^6^, ^7^

PRP plasma gel is an orthobiologic preparation obtained by controlled thermal denaturation of plasma proteins to create an albumin‐based semisolid matrix. When combined with fresh PRP, the formulation provides both regenerative potential and gradual release of bioactive molecules, resulting in prolonged biological activity compared with standard PRP.^8^, ^9^

The purpose of this technical note is to describe a simple, reproducible, and cost‐effective method for preparing PRP plasma gel and to outline its intra‐articular application in the treatment of knee OA.

## SURGICAL TECHNIQUE

The complete technique is shown in Video 1. Pearls and pitfalls of the technique are described in Table 1, and advantages and disadvantages in Table 2.

**TABLE 1:** Pearls and Pitfalls

Pearls	1. Perform the entire procedure under strict aseptic technique to minimize contamination. 2. In vacuum tubes, add heparin only after blood collection; adding beforehand may compromise the vacuum and hinder blood draw. 3. Maintain centrifugation at 800 G for 15 min for consistent separation. 4. Do not confuse RCF (G‐force) with RPM; confirm in the device manual or calculate using the rotor radius. 5. Use the formula to calculate RCF: RCF = (RPM)^2^ × 1.118 × 10^−5^ × *r* (*r* = mean rotor radius, cm); online calculators are available. 6. Online calculators are available to simplify this step: • encorbio.com • insilico.ehu.es • insilicase.com 7. Aspirate the plasma fraction close to the buffy coat for higher platelet yield. 8. Whenever possible, send PRP and plasma gel samples for laboratory platelet count. 9. Mix gel and fresh PRP multiple times through a three‐way connector to ensure homogeneity.
Pitfalls	1. Inadequate sterile technique may result in contamination or infection. 2. Incorrect heating (temperature too low or too high) compromises gel consistency and platelet function. 3. Incorrect placement of tubes in the centrifuge: tubes must be balanced, placed diametrically opposite, and filled with equal volumes to ensure stability. 4. Insufficient mixing produces a heterogeneous gel with reduced efficacy. 5. Overheating may denature platelets and reduce biologic activity. 6. Poor patient selection (advanced OA or inflammatory arthritis) may limit clinical benefit. 7. Failure to document preparation parameters reduces reproducibility between cases.

OA, osteoarthritis; PRP, platelet‐rich plasma; RCF, relative centrifugal force; RPM, rotations per minute.

**TABLE 2:** Advantages and Disadvantages

Advantages	1. Autologous, low cost, and minimally invasive. 2. Sustained release of growth factors with greater intra‐articular retention. 3. Simple, reproducible, and feasible in outpatient settings. 4. No commercial kits required, accessible even in resource‐limited systems.
Disadvantages	1. Lack of long‐term clinical data. 2. Preparation requires precise heating control. 3. Operator‐ and patient‐dependent variability. 4. Contraindicated in infection, coagulopathy, or advanced OA.

OA, osteoarthritis.

### Surgical Indications

PRP plasma gel can be indicated for patients with symptomatic knee OA who have not achieved adequate relief with conservative measures such as weight management, physical therapy, oral analgesics, or nonsteroidal anti‐inflammatory drugs. It is particularly considered for patients classified as Kellgren‐Lawrence grade II or III,^2^, ^10^ in whom surgical treatment is not yet required but symptoms significantly impair daily function or quality of life.

Although the present technical note focuses on knee OA, the formulation may be applied in other musculoskeletal conditions requiring regenerative or anti‐inflammatory support, including tendinopathies, cartilage defects, or postoperative adjunctive therapy.^8^, ^9^

### Necessary Materials for the Procedure

The preparation of PRP plasma gel requires only basic laboratory and clinical equipment, making it simple and cost‐effective. The materials required for PRP plasma gel preparation are summarized in Table 3.

**TABLE 3:** Required Materials for Platelet‐Rich Plasma Plasma Gel Preparation

**Item**	**Specification/Example**
Vacuum blood collection tubes	Sterile PET 10‐mL tubes
Anticoagulant	Heparin sodium solution (5000 IU/mL), 0.125 mL/tube
Centrifuge	Centrifuge capable of 800 G, 15 min (e.g., ONiLAB PRP Benchtop Centrifuge, ONiLAB Scientific, USA)
Incubator	Dry incubator capable of maintaining 75°C for 15 min (e.g., Vulcan PGAB Plasma Gel Incubator, Vulcan Equipamentos, Brazil)
Syringes	Sterile 5‐ and 10‐mL syringes
Connectors	Three‐way Luer Lock or dual‐lumen connectors
Aseptic materials	Sterile gloves; sterile drapes and fenestrated surgical drapes; chlorhexidine‐based antiseptic solutions (degerming and alcoholic formulations); 70% isopropyl alcohol; sterile gauze pads and compresses.
Injection needle	18G × 1½ in (40 × 12 mm) needle or equivalent

### Outpatient Setup

The procedure can be performed in an outpatient or ambulatory setting under sterile conditions. Only standard laboratory equipment is required, including a centrifuge, incubator, sterile syringes, and dual‐ or triple‐lumen syringe connectors. No commercial kits are needed, which makes this method suitable for both advanced and resource‐limited health care systems.

### Step‐by‐Step Technique

#### Step 1: PRP Preparation

**FIGURE 1:**
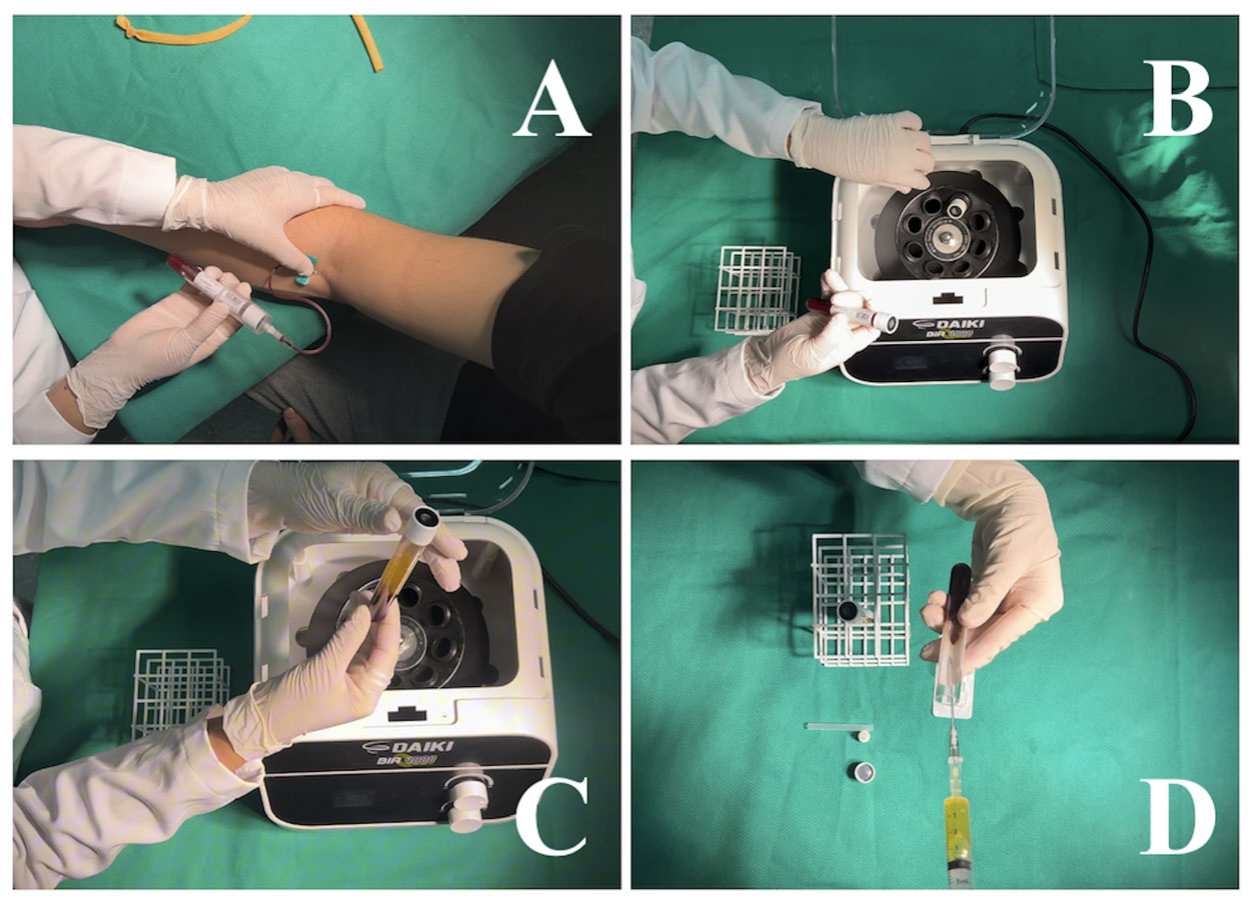
Preparation of PRP. (A) Peripheral venous blood collection into sterile vacuum tubes. (B) Placement of tubes in the centrifuge, highlighting the importance of proper balance: tubes are positioned diametrically opposite and filled with equal volumes to ensure stability during centrifugation. (C) Appearance of the centrifuged blood, showing separation of red blood cells, buffy coat, and plasma. (D) Aspiration of the plasma fraction for further processing. (PRP, platelet‐rich plasma.)

Approximately 20 mL of peripheral venous blood is collected into two 10‐mL sterile vacuum tubes (PET plastic, ionized radiation‐sterilized) without additives. Each tube receives 0.125 mL of unfractionated heparin (5000 IU/mL) to prevent coagulation. The samples are centrifuged at 800 G for 15 minutes. The lower plasma fraction and buffy coat, corresponding to PRP, are carefully aspirated (≈2.5‐3 mL from each tube) into two 5‐mL syringes and reserved (Figure 1).

#### Step 2: Albumin Gel Formation

**FIGURE 2:**
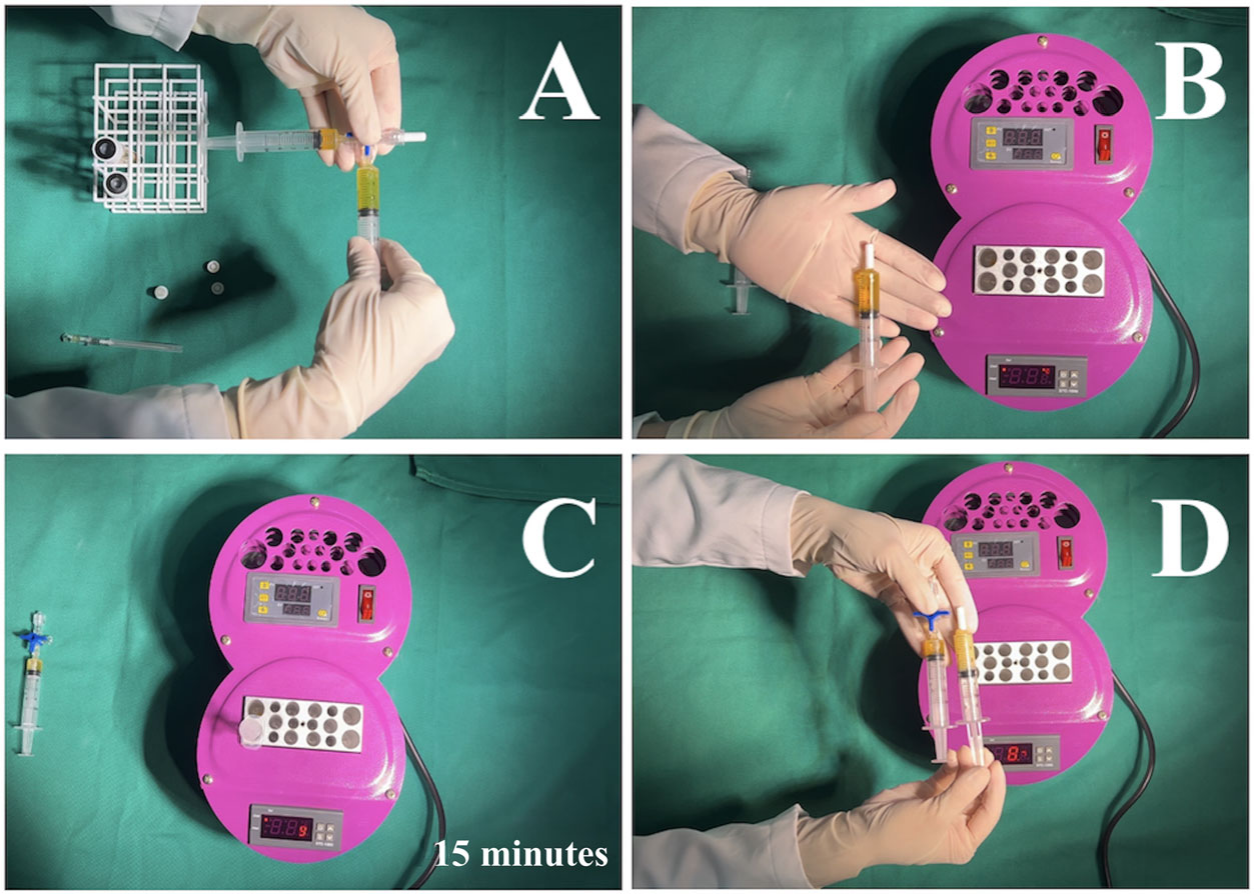
Albumin gel formation. (A) Redistribution of PRP using a three‐way Luer Lock connector in a 3:1 ratio. Approximately 3.75 mL of PRP is placed in 1 syringe, while 1.25 mL is kept in another for later use. (B,C) The larger PRP fraction is heated in an incubator at 75°C for 15 minutes, causing denaturation of albumin, fibrinogen, and other plasma proteins, and forming an albumin‐based semisolid gel. (D) The reserved fresh PRP (≈1.25 mL) and the albumin gel (≈3.75 mL) prepared for combination. (PRP, platelet‐rich plasma.)

Using a three‐way Luer Lock connector, the PRP is redistributed in a 3:1 ratio. Approximately 3.75 mL of PRP is placed in 1 syringe, while 1.25 mL is kept in another for later use. The larger PRP fraction is heated in an incubator at 75°C for 15 minutes, leading to denaturation of albumin, fibrinogen, and other plasma proteins, thereby forming an albumin‐based semisolid gel (Figure 2).

#### Step 3: PRP Plasma Gel Combination

**FIGURE 3:**
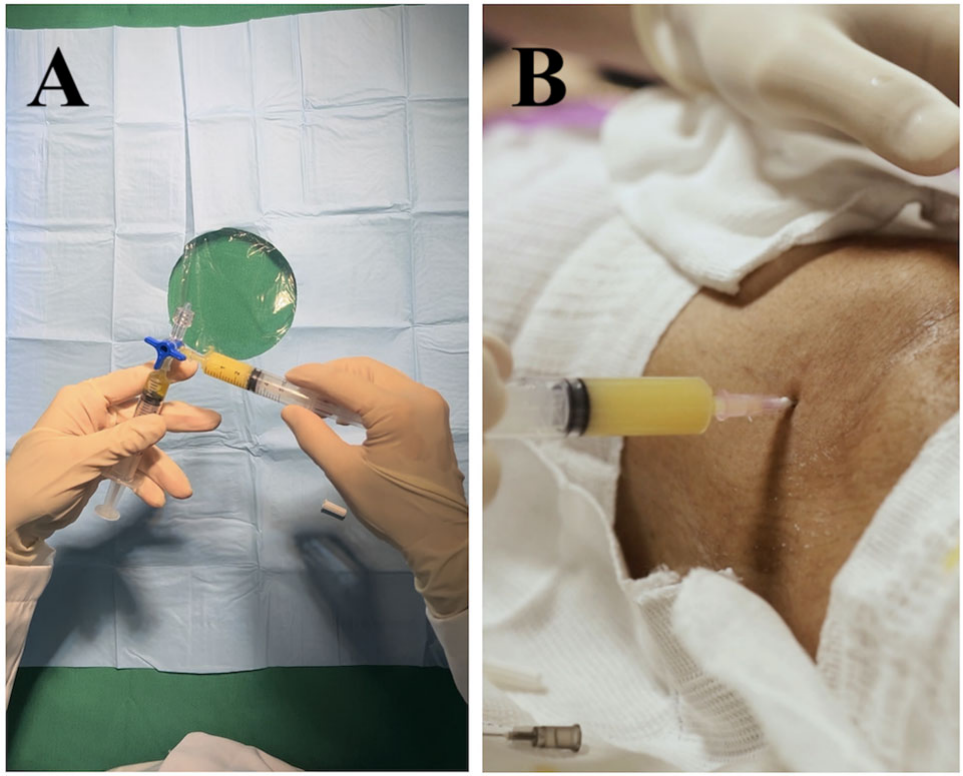
PRP plasma gel combination and application. (A) The albumin gel (≈3.75 mL) is mixed with the reserved fresh PRP (≈1.25 mL) using the three‐way connector. Multiple passes between syringes ensure homogenization, resulting in approximately 5 mL of PRP plasma gel. (B) The PRP plasma gel is transferred into a sterile syringe and injected intra‐articularly under aseptic conditions, typically through a suprapatellar or anterolateral portal of the knee.

The albumin gel obtained (≈3.75 mL) is mixed with the reserved fresh PRP (≈1.25 mL) using the three‐way connector. Multiple passes between syringes ensure homogenization, producing a final volume of approximately 5 mL of PRP plasma gel. The resulting product is stable, biocompatible, and ready for intra‐articular injection (Figure 3A).

#### Step 4: Intra‐articular Application

The PRP plasma gel is transferred to a sterile syringe and injected into the knee joint under aseptic conditions, typically through a suprapatellar or anterolateral portal (Figure 3B). The semisolid matrix facilitates local retention, potentially extending biological activity and clinical efficacy compared with conventional liquid PRP.

## DISCUSSION

PRP plasma gel represents an evolution of PRP therapy, designed to overcome the short intra‐articular half‐life of conventional liquid PRP. By creating an albumin‐based semisolid matrix that is subsequently mixed with fresh PRP, this formulation provides sustained release of growth factors and greater intra‐articular residence time, which may enhance both regenerative and anti‐inflammatory effects.^11^, ^12^, ^13^

PRP plasma gel is obtained through controlled thermal denaturation of plasma proteins, producing an albumin‐based semisolid matrix. This biomaterial has physicochemical properties that enable the sustained and gradual release of bioactive growth factors (such as TGF‐β, PDGF, and VEGF) within the intra‐articular environment, thereby prolonging therapeutic effects and enhancing tissue regeneration. Unlike conventional liquid PRP, which disperses rapidly after injection, plasma gel shows greater residence time in the target tissue, a feature that may translate into important clinical advantages.^9^, ^14^

The technique described here is simplified, reproducible, and low‐cost, relying only on basic equipment such as a centrifuge and incubator. The protocol involves heating 3 parts of PRP at 75°C for 15 minutes to form albumin gel, followed by mixing with one part of fresh, nonheated PRP using a dual‐ or triple‐lumen syringe connector. This combination results in a homogeneous, biocompatible, and stable gel ready for therapeutic use. By preserving part of the PRP in a nondenatured state, the formulation maintains cellular activity and biological viability, promoting a combined regenerative, anti‐inflammatory, and filling effect.^8^, ^9^

Compared with hyaluronic acid or corticosteroids, plasma gel offers a low‐cost, autologous, and minimally invasive option with potential for longer lasting symptom relief. The preparation is simple, reproducible, and does not require commercial kits, making it accessible in a wide range of clinical environments, including resource‐limited health care systems.^13^, ^14^, ^15^

This technique also preserves part of the PRP in its native state, maintaining cellular viability while combining the structural and biologic advantages of the gel matrix. These features may translate into improved functional outcomes and patient satisfaction.^8^, ^9^, ^16^, ^17^, ^18^

Limitations include the lack of long‐term clinical data, as current evidence is preliminary and based on short‐ to mid‐term follow‐up. Standardization of preparation protocols and comparative trials against established injectables will be essential to validate its efficacy. Additionally, as with all autologous therapies, results may vary depending on individual patient biology and preparation technique.

Overall, PRP plasma gel offers a practical, reproducible, and biologically rational method for intra‐articular injection in knee OA and may broaden the scope of orthobiologic therapies in daily practice.

## DISCLOSURES

The authors (D.A.L., R.C., C.E.S.F., Á.K.M.N., I.P.B., L.L.L., M.L.C.C.) declare that they have no known competing financial interests or personal relationships that could have appeared to influence the work reported in this paper.
